# Molecular Structure, Thermodynamic and Spectral Characteristics of Metal-Free and Nickel Complex of Tetrakis(1,2,5-thiadiazolo)porphyrazine

**DOI:** 10.3390/molecules26102945

**Published:** 2021-05-15

**Authors:** Yuriy A. Zhabanov, Alexey V. Eroshin, Igor V. Ryzhov, Ilya A. Kuzmin, Daniil N. Finogenov, Pavel A. Stuzhin

**Affiliations:** Research Institute of Chemistry of Macroheterocyclic Compounds, Ivanovo State University of Chemistry and Technology, Sheremetievskiy av. 7, 153000 Ivanovo, Russia; alexey.yeroshin@gmail.com (A.V.E.); ryzhoff.ihor@yandex.ru (I.V.R.); wonderful_37@list.ru (I.A.K.); dan.finogenof@gmail.com (D.N.F.); stuzhin@isuct.ru (P.A.S.)

**Keywords:** porphyrazine, 1,2,5-thiadiazole annulated, DFT study, CASSCF study, molecular and electronic structure, sublimation enthalpy, electronic spectra, vibrational spectra

## Abstract

The Knudsen effusion method with mass spectrometric control of the vapor composition was used to study the possibility of a congruent transition to the gas phase and to estimate the enthalpy of sublimation of metal-free tetrakis(1,2,5-thiadiazolo)porphyrazine and its nickel complex (H_2_TTDPz and NiTTDPz, respectively). The geometrical and electronic structure of H_2_TTDPz and NiTTDPz in ground and low-lying excited electronic states were determined by DFT calculations. The electronic structure of NiTTDPz was studied by the complete active space (CASSCF) method, following accounting dynamic correlation by multiconfigurational quasi-degenerate second-order perturbation theory (MCQDPT2). A geometrical structure of D_2h_ and D_4h_ symmetry was obtained for H_2_TTDPz and NiTTDPz, respectively. According to data obtained by the MCQDPT2 method, the nickel complex possesses the ground state ^1^A_1g_, and the wave function of the ground state has the form of a single determinant. Electronic absorption and vibrational (IR and resonance Raman) spectra of H_2_TTDPz and NiTTDPz were studied experimentally and simulated theoretically.

## 1. Introduction

There is a growing interest in organic materials for application in optoelectronics due to their low-cost, high-throughput film manufacturing by solution-processing techniques, high-flex-stability, easy scaling up and integration in devices [1,2,3]. Compounds based on tetrapyrrole macrocycles have interesting spectral and non-linear optical properties [4] which are practically useful for optical communication, information storage, optical switching and processing of electro-optical signals [5,6,7,8,9,10]. 

The structural formulas of the molecules discussed here are presented in the Supplementary Materials. The unsubstituted porphyrazine (or tetra-azaporphyrin) and its metallic derivatives, including the Mg(II), Ni(II) and Cu(II) complexes (MPz), were synthesized in 1952 [11]. Their tetrabenzo fused derivatives, known as phthalocyanines are so far most widely studied. Substitution of benzene rings in phthalocyanines by aromatic heterocycles, e.g., by pyrazine [12,13] or 1,2,5-chalcogenadiazole [14], have a strong impact on the electronic properties of the central porphyrazine (Pz) core which is common to these systems.

The presence of a five-membered heterocycle containing nitrogen and sulfur atoms on the periphery of tetrakis(1,2,5-thiadiazolo)porphyrazine (TTDPz) considerably modulates the physico-chemical properties of the macrocycle and its intermolecular interaction as compared to phthalocyanines [14]. Unlike phthalocyanines, or their pyrazine-fused analogues, TTDPz have no H atoms on the periphery and their molecular packing during crystal growth is determined by specific N…S interactions [15]. This is quite important for application of these phthalocyanine-type molecules as building blocks for novel functional materials in various fields [16].

Porphyrazines with fused 1,2,5-chalcogenadiazole rings having strong π-electron-deficiency [12,17], combined with their ability to form 2D layered structures in the solid state [15], can be considered as prospective n-type conducting functional materials for use in organic electronics. Thus, tetra(1,2,5-thiadiazolo)porphyrazine, H_2_TTDPz and its metal complexes MTTDPz (M = Zn(II), V(IV)O, and Fe(II)) were used as n-type organic semiconductors in prototypes of field-effect transistors and photovoltaic cells [18,19,20,21,22].

A method consisting of the precipitation of products from the thermal decomposition of highly volatile organometallic precursors (MO CVD (Metal-Organic Chemical Vapor Deposition Technique)) on single-crystal substrates has attracted increasing interest amongst methods for forming thin films. Therefore, data on the structure of molecules in the gas phase and the composition of the vapor over the investigated substances are of interest. Despite their long history, information about porphyrazines and 1,2,5-chalocogenadiazole-fused porphyrazines, especially about their structural and thermal stability properties, compared to a plethora of studies of porphyrins and phthalocyanines, is relatively scant. Awaga et al. grew single crystals of tetrakis-(thiadiazol)porphyrazine and the corresponding metal(II) derivatives, MTTDPz (M = H_2_, Fe, Co, Ni, Cu, and Zn) by sublimation under reduced pressure with continuous N_2_ gas flow and elucdated their structures by X-ray crystallographic analysis [15]. However, there are no data on the composition of the vapor. Quantum chemistry investigations of structural and optical properties in the case of transition metal complexes are often non-trivial due to the necessity to account for the multireference character of the wavefunction [23].

Theoretical DFT calculations with various basis sets have been used for the investigation of the molecular and electronic structures and spectral properties of H_2_TTDPz [24,25] and its metal complexes MTTDPz (M = Mg(II) [24,26], Ca(II) [27], Cu(II) [24,28], Fe(II) [23], Co(II) [23], Zn(II) [24,27,29], ClAl(III) and ClGa(III) [30]. Quantum-chemical calculations and interpretation of electronic and vibrational spectra were also carried out for complexes of tetra(1,2,5-thiadiazolo)porphyrazine with rare earth elements Y, La and Lu [31,32].

Vapor composition and thermodynamic and spectral characteristics are necessary to determine the possibility of using substances in the gas-phase technologies of microelectronics. The main objective of the present study is to identify the influence of the metal on the properties of macrocyclic complexes. It is important to compare the thermal stability, vapor composition, molecular structure and spectra of metal-free tetra(1,2,5-thiadiazolo)porphyrazine (H_2_TTDPz) and its nickel(II) complex (NiTTDPz). The nickel metal complex NiTTDPz is of interest because metalated porphyrazines are considered possible precursors for much sought-after three-dimensional molecular magnets and possible structures with superconducting properties [33]. Moreover, we have studied theoretically the structure of MTTDPz complexes with some transition metals [23,27] and in this work we begin an experimental study of these substances. The possibility of congruent evaporation and enthalpy of sublimation have been determined using the Knudsen effusion method with mass spectrometric control of vapor composition. The lowest excited states were also calculated in order to explain the peculiarities and tendencies observed in the experimental electronic absorption spectra. In addition, the vibrational absorption spectra were analyzed and interpreted.

## 2. Results and Discussion

### 2.1. Sublimation

It was earlier established that MTTDPz (M = H_2_, Mn, Fe, Co, Ni, Cu, and Zn) can be sublimated under vacuum [15,29], but the thermodynamic characteristics of these processes have not been reported yet.

**Figure 1:**
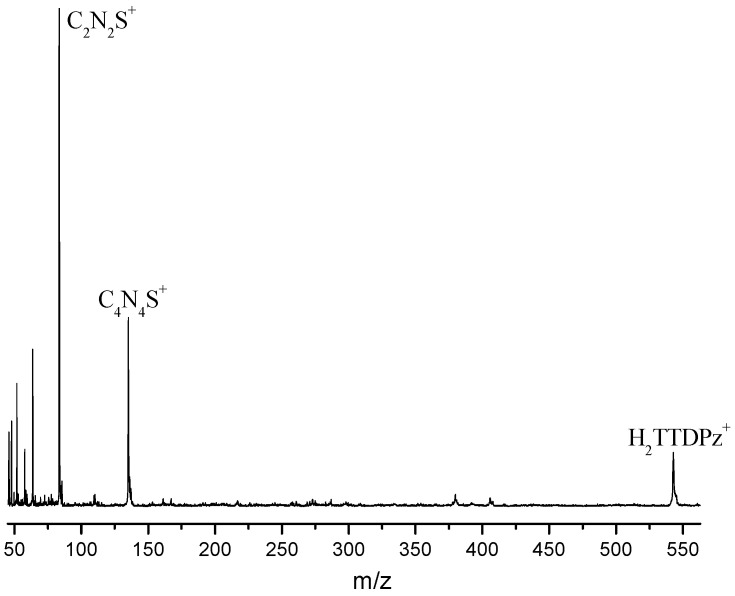
Mass spectrum of H_2_TTDPz recorded at 690 K.

The electron impact mass spectrum of H_2_TTDPz recorded at 690 K is shown in Figure 1 and the relative abundance of ions is presented in Table 1. Analyzing the mass spectrum of H_2_TTDPz, we came to the conclusion that its sublimation is accompanied by partial decomposition. The ion H_2_TTDPz^+^ has lower intensity than another heavy ion C_4_N_4_S^+^ at m/z = 136 a.m.u. The C_4_N_4_S^+^ ion corresponds to 1⁄4(TTDPz) and can be assigned to the C_4_N_4_S molecular species—1,2,5-thiadiazole-3,4-dicarbonitrile. The relative abundance of ions in mass spectrum of H_2_TTDPz recorded in the present work is similar to the relative abundance of ions in mass spectrum of ZnTTDPz recorded simultaneously with gas electron diffraction experiment [29]. However, the recording temperature of the ZnTTDPz mass spectrum was 861 K [29], which is 171 K higher than the recording temperature of the H_2_TTDPz mass spectrum.

Mass-spectrometric studies performed in connection with the sublimation process show that NiTTDPz gives a stable stream of particles at temperatures T = 632–717 K, where a molecular ion (m/z = 602) dominates, followed by several ions of weaker (3–4%) intensity. No ions corresponding to oligomeric species were detected. According to the mass spectra analysis, we are inclined to conclude that saturated vapor consists only of the parent NiTTDPz.

**Figure 2:**
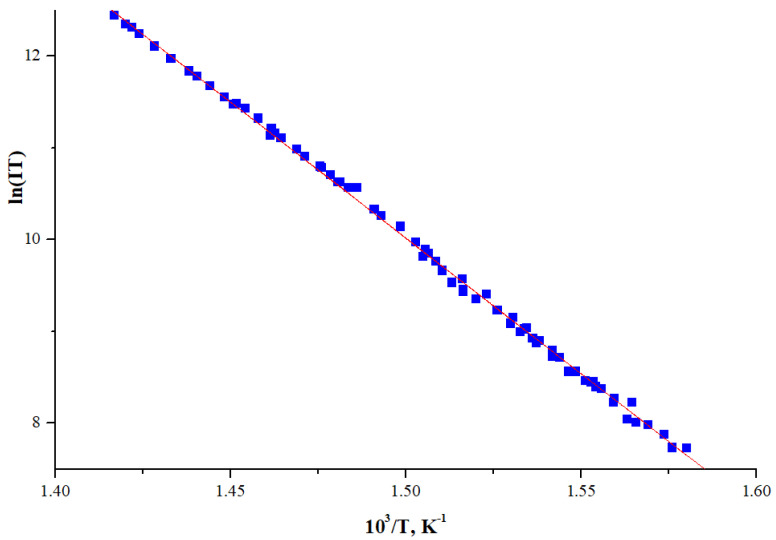
Dependence of the molecular ion intensity logarithm of NiTTDPz on temperature.

The plot of ln(IT) = f(1000/T) for NiTTDPz^+^ ion is shown in Figure 2. The set of the points is the result of measurements with a step-by-step increase in temperature, and then with a step-by-step decrease. Each point of the graph corresponds to the ion current measured after its stabilization at a given temperature. One can see that the hysteresis as the temperature increases and decreases is practically absent. This allows us to conclude that points in plots are corresponding to the equilibrium states inside the effusion cell. The dependence ln(IT) = f(1000/T) could be closely approximated by a straight line, usually observed for vaporization in the considered temperature range without change of crystallographic modification of the solid phase and without significant change of the enthalpy of vaporization. The enthalpy of sublimation value ΔH_s_ calculated by linear regression using the Clausius–Clayperon equation $\mathrm{Ln}(\mathrm{IT})=-\frac{\Delta H}{\mathrm{RT}}+C$ was found to be 246(2) kJ·mol^−1^.

### 2.2. Molecular Structure

The electronic configuration of Ni(II) is [Ar]3d^8^, and therefore it can form in ground state as either singlet or triplet complexes. Furthermore, the computational investigations in the case of Ni(II) complexes are often non-trivial due to the necessity to account for the multireference character of the wavefunction.

The electronic structure of NiTTDPz has been studied by the CASSCF method followed by an account of the dynamic electron correlation by multiconfigurational quasi-degenerate second-order perturbation theory (MCQDPT2). The compositions of the wave functions are presented in Table 2 for the low-lying electronic states. According to data obtained by the MCQDPT2 method, the NiTTDPz complex possesses the ground states ^1^A_1g_. The low-lying triplet state is higher by 88.4 kJ mol^−1^ in energy than the corresponding ground state (Table 1). It should be noted that, according to CASSCF calculations, NiTTDPz possesses a triplet ground state. Such contradictory conclusions about the multiplicity of the ground state obtained using the CASSCF and MCQDPT2 methods are apparently due to the fact that the CASSCF calculations with a small active space do not practically take into account the dynamic correlation of electrons. An analysis of the data in Table 2 shows that the wave functions of the ground states and the most low-lying triplet states have the form of a single determinant. Therefore, for the D_4h_ configuration in the electronic state ^1^A_1g,_ the geometry optimization, calculations of the force field, and vibrational and electronic spectra have been performed using the PBE0/pcseg-2 approach.

In ref [23] it was shown that crystal field theory (CFT) can be used to describe the sequence of electronic states of MPz and MTTDPz (M=Fe, Co) complexes. However, in the case of the singlet state of nickel hemi-porphyrazine [34], it is impossible to describe the sequence of electronic states using the crystal field theory. In the framework of this theory, the most energetically favorable states are those with the least repulsion between the electrons occupying the d-shell of the metal and orbitals of the macrocycle. From this point of view, the occupation of the b_2g_, e_g_, and a_1g_ MOs are the most favorable, but not b_1g_.

**Figure 3:**
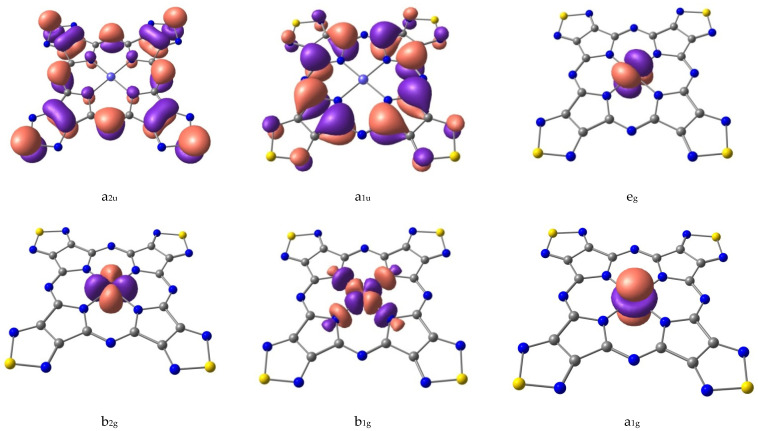
Shapes of active CASSCF molecular orbitals of NiTTDPz.

**Figure 4:**
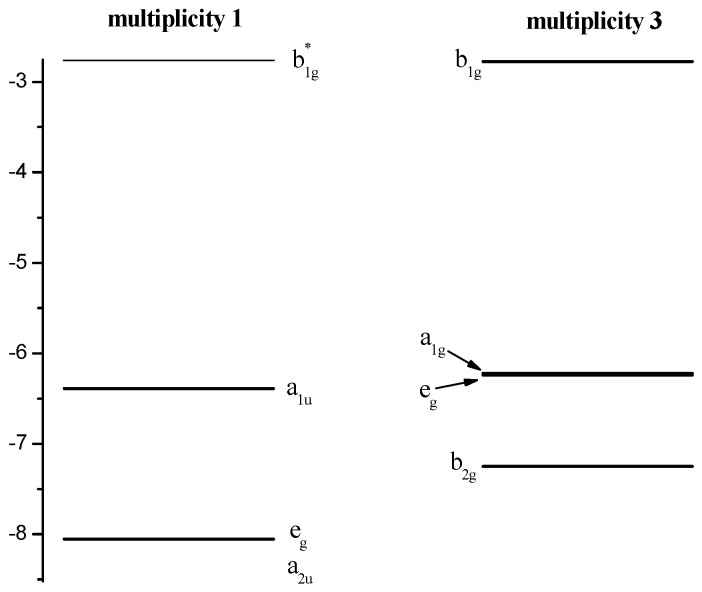
Diagram of the active energies in the CASSCF calculations of the molecular orbitals of NiTTDPz.

The crystal field theory (CFT, [35,36]) can be used to describe the sequence of NiTTDPz electronic states (Table 1) despite the fact that the shapes of two CASSCF active molecular orbitals (a_2u_ and a_1u_, Figure 3) in the singlet NiTTDPz state comprise atoms of the macrocycle rather than the metal atom. This conclusion is confirmed by the fact that the b_1g_ orbital is unoccupied in the ground state and this orbital has significantly greater energy than the other three orbitals (Figure 4).

Shapes of CASSCF active molecular orbitals (Figure 3) of triplet NiTTDPz state and their composition analysis show that the corresponding components of the d-orbitals of the metal atom make a dominant contribution. The orbitals of the macrocycle atoms are almost not involved in the formation of these molecular orbitals. The orbital of b_1g_ symmetry is an exception, since according to Figure 3 the contribution of the macrocycle orbitals can be visually observed. It should be noted that no noticeable interaction of metal d-orbitals and macrocycle orbitals was found. Thus, the crystal field theory (CFT) can be used to describe the sequence of electronic states. A diagram of the energies of active in the CASSCF calculations molecular orbitals (Figure 4) confirms this conclusion.

**Figure 5:**
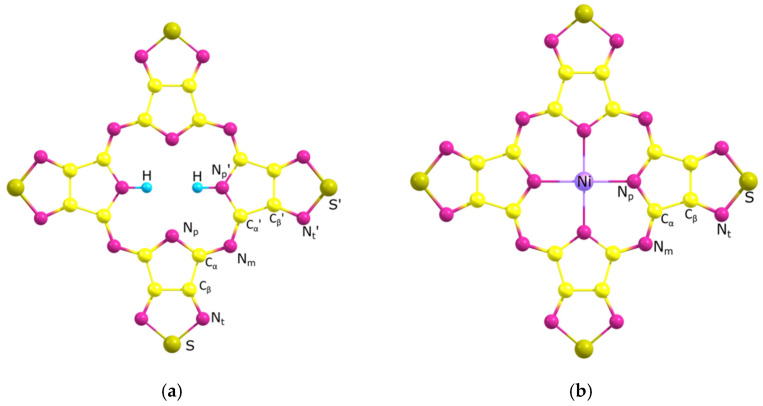
Models of H_2_TTDPz (**a**) and NiTTDPz (**b**) molecules with atom labeling.

According to DFT calculations the molecules under consideration have a planar structure with symmetry D_2h_ and D_4h_ for H_2_TTDPz and NiTTDPz, respectively (Figure 5). Note that the singlet and triplet states of the NiTTDPz molecule have significant differences in geometric structure (Table 3). In the case of triplet NiTTDPz, a significant (about 0.1 Å) increase in the (N_p_…N_p_)_opp_ and, accordingly, in N_p_-M and (N_p_…N_p_)_adj_ bond length (about 0.05 Å) distance is observed. When analyzing the data in Table 3, it can be noted that the smallest size of coordination cavity is observed for the NiTTDPz molecule in the singlet state, and the largest is for the H_2_TTDPz molecule. This confirms the conclusion of the influence of the metal on the size of the coordination cavity [23,27,32].

### 2.3. Electronic Absorption Spectra

**Figure 6:**
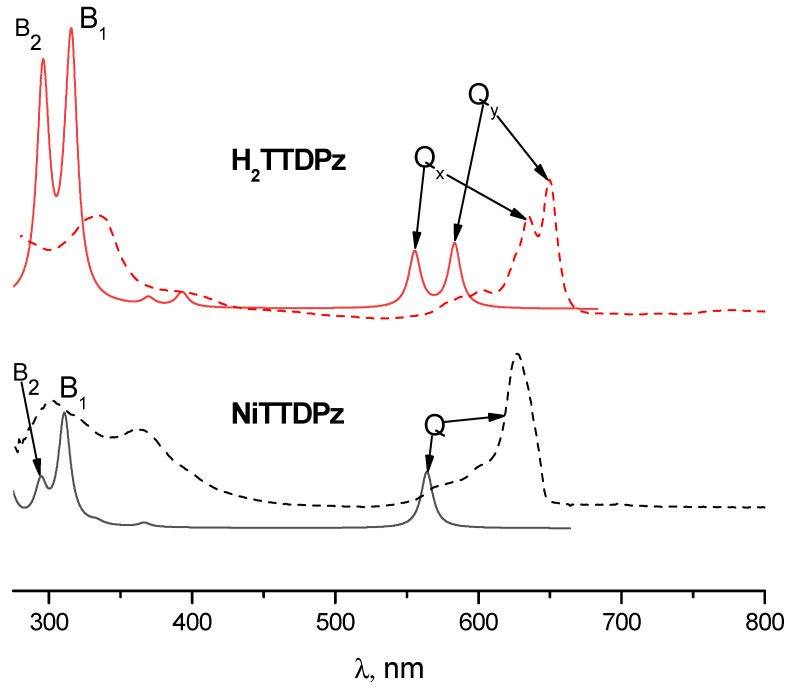
Calculated TDDFT electronic absorption spectra for H_2_TTDPz and NiTTDPz molecules (solid lines) and the corresponding experimental spectra in DMSO solutions (dashed lines).

Analyzing the electronic absorption spectra of molecules simulated by the TDDFT method, one can notice significant differences (Figure 6). The Q-band is located at about 564 nm in the spectrum of NiTTDPz, while in the spectrum of the H_2_TTDPz, splitting into two bands, Q_y_ (584 nm) and Q_x_ (554 nm), is observed. In addition to significant changes in the region of the Q-band, one can also observe a change in relative intensities in the region of the B-band (≈300–330 nm). It is clearly seen that the B_2_-band in the spectrum of NiTTDPz has a lower intensity in comparison with the B_1_-band. In the case of H_2_TTDPz, these bands are close in intensity. The obtained spectra of H_2_TTDPz and NiTTDPz can be described using the four-orbital model of Gouterman [37,38,39]; both spectra in general are quite typical for this class of compounds.

Interpretation of the electronic spectra was carried out on the basis of the results of TDDFT calculations. The calculated oscillator strengths (*f*) for the lowest excited states along with their composition (in terms of one-electron transitions) are given in Table 4 and Table 5 for H_2_TTDPz and NiTTDPz, respectively. Analysis of the data in Table 4 and Table 5 demonstrates that, in the case of the NiTTDPz complex, it can be seen that the Q-band corresponds to the transition between the frontier orbitals and the formation of the 1^1^E_u_ state. The Q_y_ and Q_x_ bands in the H_2_TTDPz spectrum correspond to transitions from the ground state to the excited electronic states 1^1^B_2u_ and 1^1^B_3u_, respectively. The 1^1^B_3u_ state is formed due to the HOMO-1→LUMO and HOMO→LUMO+1 transitions and the 1^1^B_2u_ state is formed due to the transition between the frontier orbitals. The excited states with a strong contribution to the electronic transition from the Gouterman type a_2u_ (NiTTDPz) and b_1u_ (H_2_TTDPz) orbitals, localized mainly on the inner nitrogen atoms of porphyrazine, have the highest intensity in the calculated spectra and are denoted as B band.

**Figure 7:**
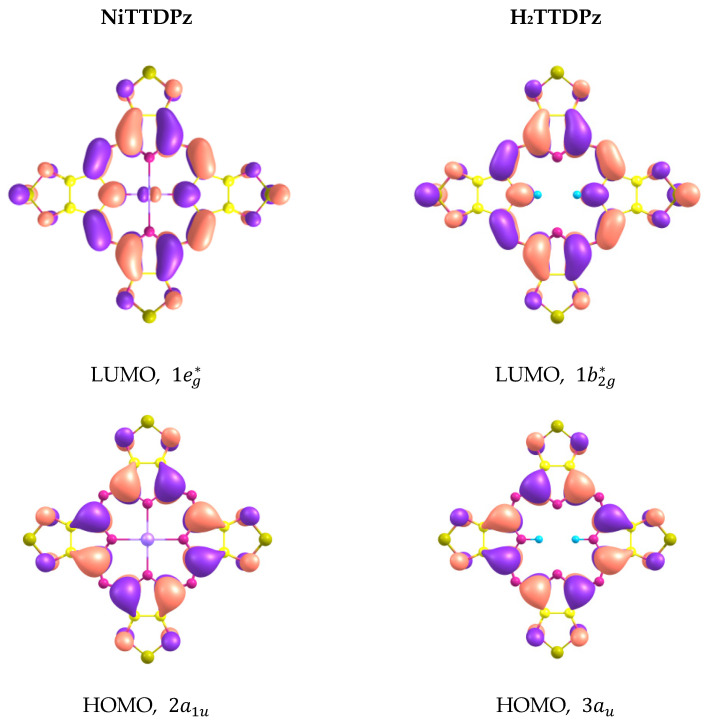

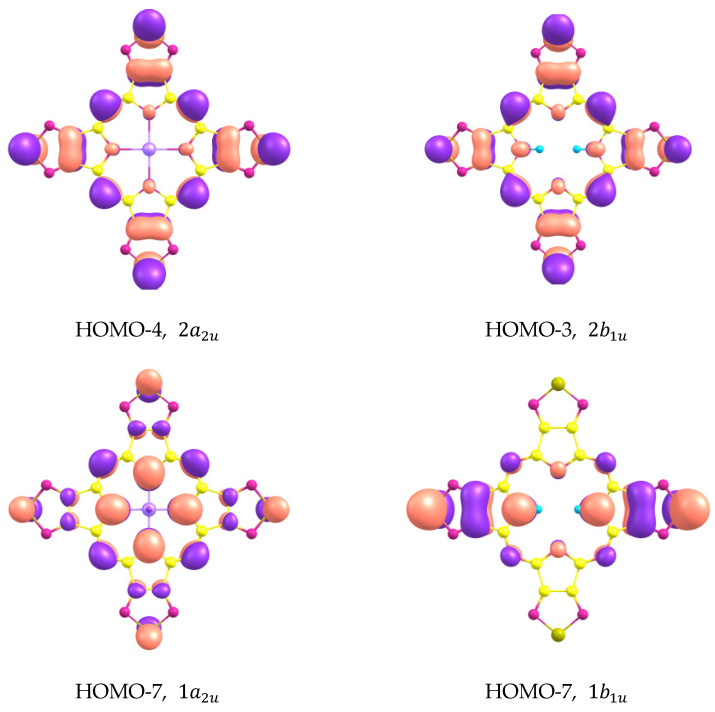
Shapes of molecular orbitals that participate in electronic transitions with large oscillator strengths.

**Figure 8:**
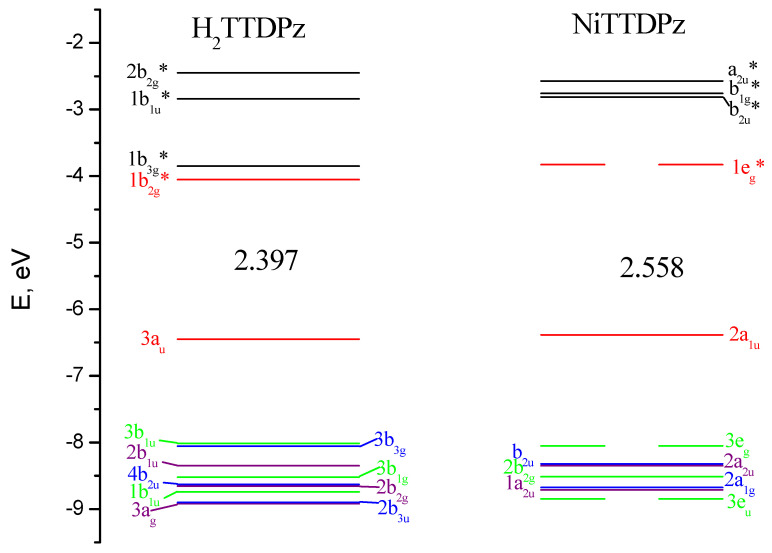
Molecular orbital (MO) level diagram for H_2_TTDPz and NiTTDPz molecules. The values of highest occupied molecular orbital–lowest unoccupied molecular orbital (HOMO–LUMO) gaps are given in eV.

The shapes of molecular orbitals (MOs) that participate in electronic transitions with large oscillator strengths are shown in Figure 7 and the energy diagram of molecular orbitals for H_2_TTDPz and NiTTDPz is shown in the Figure 8. The symmetry of the frontier MOs in the NiTTDPz complex is also typical for metal complexes of porphyrazines and tetrakis(1,2,5-thiadiazolo)porphyzarines—a_1u_ for HOMO and a pair of degenerate orbitals e_g_^*^ for LUMO [26,27,32,40,41]. The shapes of HOMO orbitals for H_2_TTDPz and NiTTDPz are similar, despite different types of symmetry. The b_2g_^*^ orbital (LUMO) of the H_2_TTDPz molecule is a linear combination of bonding orbitals along the C_α_-C_β_ and N_m_-C_α_ bonds, as well as antibonding orbitals predominantly belonging to N_t_ atoms and pairs of N_p_ and S atoms. In the case of NiTTDPz, the shape of the 1e_g_^*^ orbital can be characterized in the same way, but the contribution of the d-orbital of the nickel atom is noticeable. In addition to the frontier orbitals, HOMO-3 (H_2_TTDPz), HOMO-4 (NiTTDPz), and HOMO-7 of both compounds are involved in a large number of electronic transitions. Analyzing the composition of the HOMO-3 and HOMO-4 orbitals of the corresponding molecules, one can note the fact that, despite different symmetry (b_3u_ and a_2u_, respectively), these MOs are practically identical in composition and only slightly differ in the regions of the N_p_ atoms.

The HOMO-LUMO gap is 2.40 eV for H_2_TTDPz and 2.56 eV for NiTTDPz, respectively. It should be noted that this value is typical for unsubstituted porphyrazines, but falls outside the range of previously studied thiadiazol annulated porphyrazines (2.24–2.29 eV for MTTDPz (M = Mg, Ca, Zn, Y, La, Lu) [26,27,32]).

### 2.4. Vibrational Spectra

**Figure 9:**
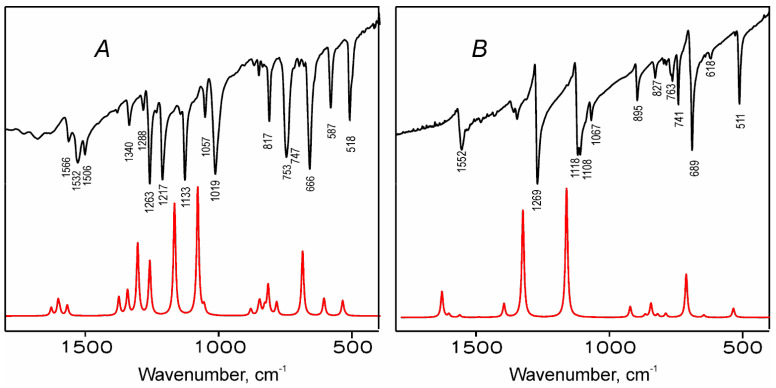
Comparison of the experimental (black lines) and simulated (red lines) IR spectra of H_2_TTDPz (**A**) and NiTTDPz (**B**).

Theoretical results were also used for interpretation of the experimental vibrational spectra of H_2_TTDPz and NiTTDPz. The IR and Raman spectra were simulated on the basis of the normal mode frequencies and band intensities, which have been calculated by the DFT (PBE0/pcseg-2) method in a harmonic approximation. Description of the main IR active vibrations is presented in Table 6. As can be seen from Figure 9, the simulated IR spectra have fairly good correspondence with the experimental spectra and theoretical data can be used for assignment of the most intense bands.

The IR spectrum of NiTTDPz contains fewer relatively intense peaks compared to H_2_TTDPz. The latter has bands with similar frequency values, but different intensities, which can be explained by its lower symmetry in contrast to NiTTDPz, where the vibrational transitions are degenerate. Thereby we can conclude that the metal introduction decreases the number of strong and medium-strong bands particularly in the 1000–1500 cm^−1^ region. Furthermore, these bands are mostly contributed by stretching vibrations in the case of NiTTDPz, while bending predominates in H_2_TTDPz.

The two most intensive bands, located for NiTTDPz in the 1100–1350 cm^−1^ region, correspond to skeletal vibrations of the macrocycle with a predominant contribution of the N_p_-C_α_ stretching vibrations. For H_2_TTDPz four intense bands are present in this region and have comparable contribution from the stretching vibrations of the N_p_-C_α_ and C_α_-C_β_ bonds in the pyrrole and pyrrolenine rings and in-plane deformation modes. With increasing frequency, a decrease of the out-of-plane vibrations contribution is observed. The ratio of stretching vibrations at the periphery also increases. One strong band is present for NiTTDPz and three less intense bands for H_2_TTDPz in the 1500–1650 cm^−1^ region. These bands make a considerable contribution to the stretching vibrations of the bridging N_m_-C_α_ bonds, C_α_-C_β_ bonds in pyrrole rings and C_β_-N_t_ bonds in the fused heterocycle. The out-of-plane deformations of 1,2,5-thiadiazole rings appear as medium-strong bands at 500–550 cm^−1^. Calculations predict a medium peak of N_p_-H stretching at 3554 cm^−1^. In the experimental spectrum, this vibration is observed at lower frequencies 3291 cm^−1^, evidencing existence of strong intramolecular hydrogen bonding the center of macrocycle.

**Figure 10:**
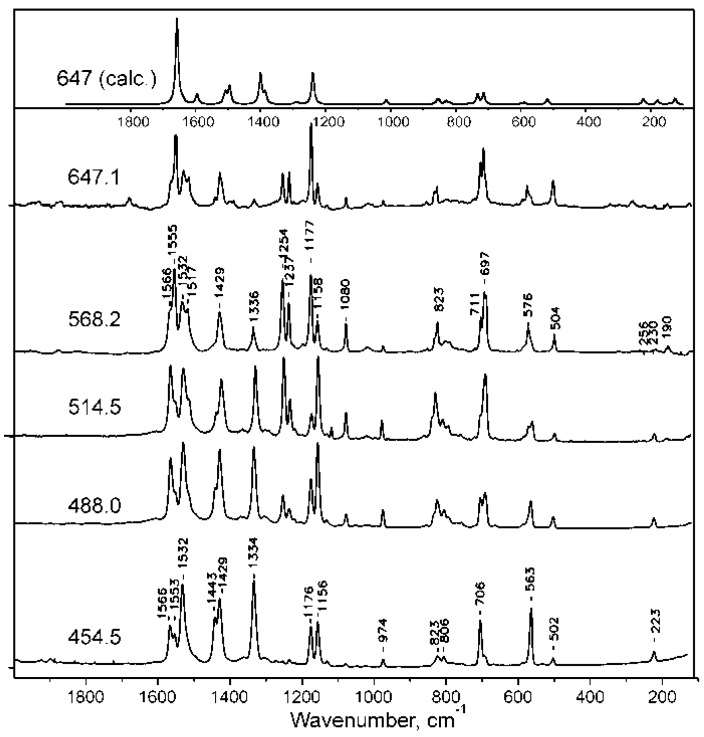
Resonance Raman spectra of H_2_TTDPz at different excitations (454.5, 514.5, 568.4, 647.1) and the theoretical spectrum representing Raman active vibrational modes.

**Figure 11:**
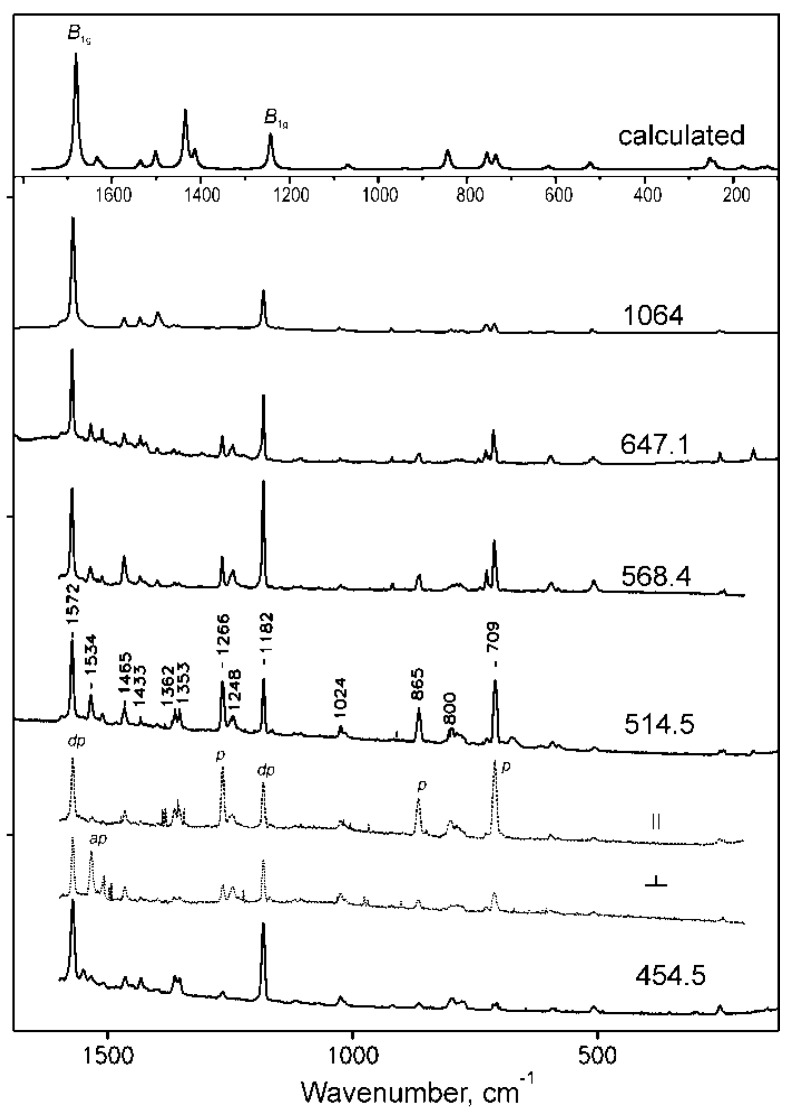
Resonance Raman spectra of NiTTDPz at different excitations (454.5, 514,5, 568.4, 647.1 and 1064 nm) and the theoretical spectrum representing Raman active vibrational modes. Polarized spectra are shown for 514.5 nm.

The resonance Raman spectra of NiTTDPz and H_2_TTDPz obtained on rotating KBr disks using various excitation wavelengths at 80 K are displayed in Figure 10 and Figure 11 along with the theoretical spectra representing Raman active vibrational modes. The description of the Raman vibrations is presented in Table 7. The calculated spectral patterns are in reasonable agreement with the experimental spectra, although the position of some bands varies by up to 100 cm^−1^. Nevertheless, the theoretical data on the contribution of the vibrations of different fragments of the macrocycle to the normal modes are quite useful in the assignment of the experimental spectra.

In the Raman spectra of NiTTDPz recording using excitation in the virtual state (λ_ex_ = 1064 nm), two intense bands are observed at 1572 and 1182 cm^−1^. They are also dominant in the resonance Raman spectra recorded upon excitation at 454.5 and 647.2 nm, i.e., in the region of the absorption bands of the two lowest electronic ππ* transitions. These depolarized bands can be assigned to the non-totally symmetric B_1g_ and B_2g_ modes which are calculated at 1681 and 1242 cm^−1^ and involve skeletal vibrations of the central porphyrazine core with strong contribution from the N_m_-C_α_ and N_p_-C_α_ bonds formed by meso- and pyrrolic nitrogen atoms, respectively. In the spectrum obtained using excitation between the Soret and Q bands (λ_ex_ = 514.5 nm), along with these depolarized bands several additional bands are enhanced. The polarized bands at 708, 865 and 1266 cm^−1^ should originate from totally symmetric modes. Indeed, calculations predict A_1g_ vibrations involving atoms constituting fused 1,2,5-thiadiazole rings at 735, 844 and 1413 cm^−1^. The appearance of the anomalously polarized band at 1534 cm^−1^ might indicate the enhancement of the A_2g_ type vibration. Evidently, A_1g_ and A_2g_ vibrations are enhanced due to vibronic coupling with weak electronic transitions 2^1^E_u_ and 3^1^E_u_ (Table 5), which should be present near λ_ex_ = 514.5 nm.

The metal free macrocycle H_2_TTDPz, due to the presence of two pyrrole and two pyrrolenine type fragments, has lower D_2h_ symmetry and its resonance Raman spectra (see Figure 10) are richer than for NiTTDPz (D_4h_). It can be seen that intense vibrations of the macrocyclic skeleton appearing as single bands for NiTTDPz are split into two components in the experimental spectra of H_2_TTDPz (1572 → 1566 and 1555; 1534 → 1532 and 1517; 1182 → 1177 and 1158; 709 → 711 and 697 cm^−1^). The intensity ratio of the components is dependent on the excitation wavelength. When excitation is shifted from the Q- to Soret band region, the bands containing a considerable contribution from the vibrational modes of the fused 1,2,5-thiadiazole rings are enhanced. This is not surprising since the Q-band transition is mainly localized on the atoms of the central macrocyclic core, while molecules with strong participation from the fused heterocycles participate in the electronic transitions in the Soret band region. Another remarkable feature in the experimental spectra of H_2_TTDPz is high intensity of the bands at 1430–1440, 1334 and 563 cm^−1^, especially at the excitation at 454.5 nm.

## 3. Materials and Methods

### 3.1. Experimental Details

H_2_TTDPz was synthesized from the lithium(I) complex Li_2_TTDPz as described earlier [40]. NiTTDPz was prepared from H_2_TTDPz and nickel(II) acetate in DMSO at 100 °C following previously published methodology [42].

UV/Vis absorption spectra were recorded on a Cary 60 spectrometer. Resonance Raman spectra were recorded using Dilor XY multi-channel spectrometer and excitation by Ar^+^ and Kr^+^ lasers (Spectra Physics) for samples in KBr pellets at 80 K. FT Raman spectra were measured using IFX 66 CS/FRA 107 Bruker interferometer and excitation by NdYAG Atlas laser (Type 300, 1064 nm) at 10 K.

The sublimation of H_2_TTDPz and NiTTDPz has been investigated by Knudsen method using the MI-1201 commercial magnetic sector mass spectrometer adapted to thermodynamic studies and described in detail in [43,44]. The solid samples were sublimated from a stainless steel effusion cell. The ratio of the cross-sectional square of the cell to the square of the effusion orifice was about 1000, which made it possible to practically eliminate the violation of thermodynamic equilibrium due to the efflux of vapors from the effusion orifice in the experiments performed. The cell temperature was measured by a tungsten-rhenium thermocouple W-Re 5/20. The energy of ionizing electrons was 70 eV, accelerating voltage 5 kV, and cathode emission current I_emis_ of 0.5 mA.

### 3.2. Computational Details

The electronic structure of NiTTDPz has been studied by the CASSCF method followed by accounting for dynamic electron correlation by multiconfigurational quasi-degenerate second-order perturbation theory (MCQDPT2). Eight electrons in five molecular orbitals consisting mainly of the 3d orbitals of Ni atom were selected for the active space. The doubly occupied orbitals corresponding to the 1s orbitals of C, N, S, and Ni and the 2s and 2p orbitals of S and Ni were frozen in the MCQDPT2 calculations. The triple-zeta basis sets pcseg-2 [45] from the Basis Set Exchange database [46,47] were used in all calculations.

DFT/PBE0-based investigations of H_2_TTDPz and NiTTDPz included geometry optimizations followed by computations of harmonic vibrations and TDDFT calculations of the electronic absorption spectra. The number of the calculated excited states was 30. The applicability of PBE0 functional for first row transition metal systems was shown by Jensen et al. [48]. All calculations were performed using the Firefly QC package [49], which is partially based on the GAMESS (US) [50] source code. The molecular models and orbitals demonstrated in the paper were visualized by means of the Chemcraft program [51]. Optimized Cartesian coordinates of H_2_TTDPz and NiTTDPz are available in the Supplementary Materials.

Description of the vibrational modes is carried out based on the analysis of the distribution of the potential energy of normal vibrations by natural vibrational coordinates. This analysis was performed using the VibModule program [52].

## 4. Conclusions

Based on mass spectrometric investigations, it was established that NiTTDPz forms a stable stream of particles and enthalpy of sublimation was estimated using the Clausius—Clayperon equation. However, analysis of the mass spectrum showed that sublimation of H_2_TTDPz is accompanied by partial decomposition.

It has been found that H_2_TTDPz and NiTTDPz have a planar macrocycle structure of D_2h_ and D_4h_ symmetry, respectively. The complexes of nickel NiTTDPz possess the ground state ^1^A_1g_ and the wave function has the form of a single determinant. It has been shown that for the studied complex NiTTDPz the crystal field theory (CFT) can be used to describe the sequences of the electronic states.

The effect of the introduction of the metal was studied on the basis of the results of TDDFT calculations. The electronic spectra of investigated molecules can be described by the four-orbital Gouterman model.

It has been shown that the theoretical data on the contribution of the vibrations of different fragments of the macrocycle to the normal modes are useful in the assignment of the experimental IR and Raman spectra.

## Figures and Tables

**Table 1:** Relative abundance of ions from H_2_TTDPz at 690 K.

Ion	*m*/*z*	Intensity, %
H_2_TTDPz^+^	546	11
C_4_N_4_S^+^	136	38
C_2_N_2_S^+^	84	100
S_2_^+^	64	32
C_2_N_2_^+^	52	25

**Table 2:** The relative energies (kJ/mol) of exited states and contributions (in %) of electronic configurations to the wave functions from MCQDPT2 calculations.

State	Contributions	ΔE, kJ/mol
^1^A_1g_	96/(a_2u_)^2^(a_1u_)^2^(e_g_)^4^(b_1g_)^0^/	0.0
^1^E_g_	99/(a_2u_)^2^(a_1u_)^2^(e_g_)^3^(b_1g_)^1^/	263.0
^1^E_u_	97/(a_2u_)^2^(a_1u_)^2^(e_g_)^2^(b_1g_)^2^/	578.1
^1^A_1g_	97/(a_2u_)^2^(a_1u_)^2^(e_g_)^2^(b_1g_)^2^/	581.5
^1^E_u_	96/(a_2u_)^2^(a_1u_)^2^(e_g_)^2^(b_1g_)^2^/	737.2
^3^B_1g_	100/(b_2g_)^2^(e_g_)^4^(a_1g_)^1^(b_1g_)^1^/	82.3
^3^E_g_	93/(b_2g_)^2^(e_g_)^3^(a_1g_)^2^(b_1g_)^1^/	92.6
^3^A_2g_	30/(b_2g_)^2^(e_g_)^2^(a_1g_)^2^(b_1g_)^2^/+70/(b_2g_)^1^(e_g_)^4^(a_1g_)^2^(b_1g_)^1^/	174.8
^3^E_g_	32/(b_2g_)^1^(e_g_)^3^(a_1g_)^2^(b_1g_)^2^/+47/(b_2g_)^1^(e_g_)^3^(a_1g_)^2^(b_1g_)^2^/+16/(b_2g_)^2^(e_g_)^3^(a_1g_)^1^(b_1g_)^2^/	331.0
^3^B_2g_	100/(b_2g_)^1^(e_g_)^4^(a_1g_)^1^(b_1g_)^2^/	338.5

**Table 3:** Internuclear distances (r, in Å) and valence angles (∠, in deg.) of H_2_TTDPz and NiTTDPz.

	H_2_TTDPz PBE0/Pcseg-2	NiTTDPz (^1^A_1g_) PBE0/Pcseg-2	NiTTDPz (^3^B_1g_) PBE0/Pcseg-2	H_2_TTDPz [15] X-ray *	NiTTDPz [15] X-ray *
N_p_-M/N_p_’-M	2.233/1.011	1.927	1.976	2.281/0.891	1.922
N_p_-C_α_/N_p_’-C_α_’	1.359/1.375	1.374	1.364	1.371/1.382	1.385
C_α_-N_m_/C_α_’-N_m_	1.320/1.301	1.303	1.313	1.331/1.311	1.313
C_α_-C_β_/C_α_’-C_β_’	1.459/1.447	1.446	1.464	1.464/1.445	1.449
C_β_-C_β_/C_β_’-C_β_’	1.408/1.416	1.401	1.407	1.400/1.408	1.396
C_β_-N_t_/C_β_’-N_t_’	1.313/1.317	1.317	1.310	1.326/1.329	1.326
N_t_-S/N_t_’-S’	1.631/1.622	1.626	1.634	1.646/1.631	1.640
(N_p_…N_p_)/(N_p_’…N_p_’)	3.958/4.092	3.854	3.952	3.952/4.062	3.844
(N_p_…N_p_’)	2.847	2.725	2.794	2.837	2.718
∠ (MN_p_’C_α_)	122.7	125.7	124.9	122.8	125.9
∠ (N_p_C_α_N_m_)/(N_p_’C_α_’N_m_)	128.2/128.8	128.4	128.2	128.1/129.8	128.7
∠ (C_α_N_m_C_α_’)	125.0	122.0	123.8	123.5	120.7
∠ (C_α_N_p_C_α_)/(C_α_’N_p_C_α’_)	109.3/114.7	108.7	110.2	108.7/113.6	108.1
∠ (N_t_SN_t_)/(N_t_’S’N_t_’)	100.7/101.3	101.1	100.4	100.5/101.3	100.6

* X-ray crystallographic data for H_2_TTDPz and α—NiTTDPz. Because of distortion of the structure in the crystals, the parameters presented in the table are average values.

**Table 4:** Calculated composition of the lowest excited states and corresponding oscillator strengths for H_2_TTDPz molecule.

State	Composition (%)	λ (nm)	*f*	Exp λ (nm)
1^1^B_2u_	$3a_{u}\to 1b_{2g}^{*}\left(91\right)$	583	0.32	650
1^1^B_3__u_	$2b_{1u}\to 1b_{2g}^{*}\left(7\right)$ $3a_{u}\to 1b_{3g}^{*}\left(87\right)$	556	0.28	635
2^1^B_3__u_	$3b_{1u}\to 1b_{2g}^{*}\left(90\right)$	393	0.07	
4^1^B_3__u_	$1b_{1u}\to 1b_{2g}^{*}\left(12\right)$ $2b_{1u}\to 1b_{2g}^{*}\left(65\right)$ $3a_{u}\to 1b_{3g}^{*}\left(7\right)$ $3a_{u}\to 2b_{3g}^{*}\left(7\right)$	316	1.21	335
4^1^B_2u_	$1b_{1u}\to 1b_{3g}^{*}\left(56\right)$ $2b_{1u}\to 1b_{3g}^{*}\left(41\right)$	312	0.18	
5^1^B_3__u_	$1b_{1u}\to 1b_{2g}^{*}\left(84\right)$ $2b_{1u}\to 1b_{2g}^{*}\left(7\right)$	298	0.15	
5^1^B_2u_	$1b_{1u}\to 1b_{3g}^{*}\left(37\right)$ $2b_{1u}\to 1b_{3g}^{*}\left(42\right)$ $3a_{u}\to 1b_{2g}^{*}\left(5\right)$ $3a_{u}\to 2b_{2g}^{*}\left(6\right)$	296	1.03	
8^1^B_2u_	$1a_{u}\to 1b_{2g}^{*}\left(10\right)$ $2a_{u}\to 1b_{2g}^{*}\left(29\right)$ $2b_{3g}\to 2b_{1u}^{*}\left(32\right)$ $3b_{1u}\to 2b_{3g}^{*}\left(27\right)$	242	0.57	
8^1^B_3__u_	$1a_{u}\to 1b_{3g}^{*}\left(14\right)$ $2a_{u}\to 1b_{3g}^{*}\left(15\right)$ $1b_{1u}\to 2b_{2g}^{*}\left(7\right)$ $2b_{2g}\to 1b_{1u}^{*}\left(61\right)$	237	0.31	
9^1^B_3__u_	$1b_{1u}\to 2b_{2g}^{*}\left(5\right)$ $2b_{2g}\to 1b_{1u}^{*}\left(6\right)$ $2b_{1u}\to 2b_{2g}^{*}\left(7\right)$	234	0.11	
11^1^B_3__u_	$1a_{u}\to 1b_{3g}^{*}\left(13\right)$ $1b_{1u}\to 2b_{2g}^{*}\left(41\right)$ $2b_{2g}\to 1b_{1u}^{*}\left(18\right)$ $2b_{2g}\to 2b_{1u}^{*}\left(28\right)$	215	0.08	

**Table 5:** Calculated composition of the lowest excited states and corresponding oscillator strengths for NiTTDPz molecule.

State	Composition (%)	λ (nm)	*f*	Exp λ (nm)
1^1^E_u_	$2a_{1u}\to 1e_{g}^{*}$ (93)	564	0.28	627
2^1^E_u_	$2a_{1u}\to 2e_{g}^{*}$ (94)	367	0.02	
3^1^E_u_	$1a_{2u}\to 1e_{g}^{*}$ (7) $b_{2u}\to 1e_{g}^{*}$ (86)	333	0.02	
4^1^E_u_	$1a_{2u}\to 1e_{g}^{*}$ (8) $2a_{2u}\to 1e_{g}^{*}$ (78) $b_{2u}\to 1e_{g}^{*}$ (8)	311	0.55	362
5^1^E_u_	$1a_{2u}\to 1e_{g}^{*}$ (76) $2a_{2u}\to 1e_{g}^{*}$ (8)	294	0.19	
6^1^E_u_	$3e_{g}\to b_{2u}^{*}$ (97)	269	0.31	
7^1^E_u_	$b_{1u}\to 1e_{g}^{*}$ (6) $3e_{g}\to a_{2u}^{*}$ (89)	257	0.26	
8^1^E_u_	$b_{1u}\to 1e_{g}^{*}$ (5) $3e_{u}\to b_{1g}^{*}$ (88)	244	0.07	
9^1^E_u_	$b_{1u}\to 1e_{g}^{*}$ (83) $3e_{u}\to b_{1g}^{*}$ (7) $3e_{g}\to a_{2u}^{*}$ (7)	240	0.34	
10^1^E_u_	$1a_{1u}\to 1e_{g}^{*}$ (16) $b_{2u}\to 1e_{g}^{*}$ (69)	231	0.05	
13^1^E_u_	$1a_{1u}\to 1e_{g}^{*}$ (13) $2e_{g}\to b_{2u}^{*}$ (11) $1a_{2u}\to 2e_{g}^{*}$ (48) $2a_{2u}\to 2e_{g}^{*}$ (10) $b_{2u}\to 2e_{g}^{*}$ (5) $3e_{g}\to b_{1u}^{*}$ (10)	217	0.07	

**Table 6:** Assignment of the IR vibrations of the H_2_TTDPz and NiTTDPz molecules.

Frequency, cm^−1^	I_rel_, %	Symmetry	Assignment *	Exp, cm^−1^
H_2_TTDPz				
535 (ω_34_)	12	B_3u_	OPB (N_t_-C_α_-C_β_-C_β_) (23), θ (C_β_-N_t_-S-N_t_) (36)	518
607 (ω_44_)	14	B_2u_	r(C_α_-C_β_) (17), r(N_t_-S) (11), φ(C_β_-N_t_-S) (18), φ(N_t_-S-N_t_) (19)	587
686 (ω_44_)	29	B_1u_	φ(N_p_-C_α_-C_β_) (7), φ(C_α_-N_p_’-C_α_) (6), φ(N_m_-C_α_-C_β_) (10), φ(C_α_-C_β_-C_β_) (8), φ(C_β_-C_β_-N_t_) (8), φ(C_β_-N_t_-S) (6)	666
687 (ω_45_)	22	B_2u_	φ(C_α_-N_p_-C_α_) (6), φ(N_p_-C_α_-C_β_) (10), φ(N_p_’-C_α_-C_β_) (8), φ(N_m_-C_α_-C_β_) (11), φ(C_α_-C_β_-C_β_) (10), φ(C_β_-C_β_-N_t_) (8), φ(C_β_-N_t_-S) (6)	666
785 (ω_54_)	10	B_2u_	r(N_t_-S) (5), φ(N_p_-C_α_-N_m_) (5), φ(N_p_’-C_α_-N_m_) (13), φ(C_α_-N_p_’-H) (10),φ(C_α_-N_m_-C_α_) (12), φ(N_m_-C_α_-C_β_) (11),	753
817 (ω_59_)	24	B_3u_	OPB (H-C_α_-C_α_-N_p_’) (24), OPB (C_β_-N_p_-N_m_-C_α_) (11),θ (C_α_-H-N_p_-C_α_-N_m_-C_β_) (24), θ (C_α_-N_m_-C_α_-N_p_-C_β_) (8), θ (C_α_-N_m_-C_α_-N_p_’-C_β_) (8)	817
1083 (ω_75_)	100	B_1u_	r(N_p_-C_α_) (13), r(N_m_-C_α_) (12), r(C_α_-C_β_) (8), φ(N_p_-C_α_-C_β_) (8), φ(N_m_-C_α_-C_β_) (8), φ(C_α_-C_β_-C_β_) (8), φ(C_β_-C_β_-N_t_) (6)	1019
1171 (ω_78_)	87	B_2u_	r(N_p_’-C_α_) (9), r(N_m_-C_α_) (6), r(C_α_-C_β_) (7), φ(C_α_-N_p_’-H) (14), φ(N_p_’-C_α_-N_m_) (5), φ(N_m_-C_α_-C_β_) (8), φ(C_α_-N_m_-C_α_) (6), φ(C_β_-C_β_-N_t_) (7), φ(C_β_-N_t_-S) (6)	1133
1264 (ω_81_)	42	B_2u_	r(N_p_-C_α_) (5), r(N_p_’-C_α_) (6), φ(C_α_-N_p_-C_α_) (6), φ(N_p_-C_α_-N_m_) (8), φ(C_α_-N_p_’-H) (26)	1217
1310 (ω_85_)	56	B_1u_	r(N_p_’-C_α_) (5), r(C_α_-C_β_) (7), r(C_β_-C_β_) (5), φ(N_p_’-C_α_-N_m_) (7), φ(N_p_-C_α_-N_m_) (7), φ(C_α_-C_β_-N_t_) (9)	1263
1348 (ω_86_)	19	B_2u_	r(N_p_-C_α_) (9), φ(C_α_-N_p_-C_α_) (5), φ(N_p_-C_α_-N_m_) (11), φ(C_α_-N_p_’-H) (28), φ(N_m_-C_α_-C_β_) (9), φ(C_α_-N_m_-C_α_) (7)	1288
1381 (ω_88_)	14	B_2u_	r(C_α_-C_β_) (7), r(C_β_-C_β_) (7), φ(C_α_-N_p_’-H) (15), φ(C_α_-C_β_-N_t_) (9), φ(C_β_-C_β_-N_t_) (7), φ(C_β_-N_t_-S) (6)	1340
1577 (ω_98_)	9	B_1u_	r(N_p_-C_α_) (5), r(N_m_-C_α_) (53), r(C_α_-C_β_) (9), r(C_β_-N_t_) (12)	1506
1611 (ω_103_)	12	B_1u_	r(N_m_-C_α_) (5), r(C_α_-C_β_) (15), r(C_β_-N_t_) (13), φ(C_α_-C_β_-C_β_) (14), (C_β_-C_β_-N_t_) (14)	1532
1637 (ω_104_)	7	B_2u_	r(N_m_-C_α_) (34), r(C_α_-C_β_) (23), r(C_β_-N_t_) (16), φ(C_α_-N_p_’-H) (6)	1566
3554 (ω_107_)	45	B_1u_	r(N_p_’-H) (69), r(N_p_’-C_α_) (5), φ(C_α_-N_p_’-C_α_) (6), φ(C_α_-N_p_’-H) (6), φ(C_α_-N_p_’-N_m_) (6), φ(N_p_’-C_α_-C_β_) (6)	3291
NiTTDPz				
533 (ω_35_)	7	A_2u_	θ (N_m_-C_α_-C_β_-N_t_) (29), θ (C_β_-N_t_-S-N_t_) (22), θ(C_α_-N_m_-C_α_-N_p_-C_β_) (32)	511
711 (ω_46_–ω_47_)	33	E_u_	r(N_p_-Ni) (10), r(N_m_-C_α_) (12), r(C_α_-C_β_) (5), φ(C_α_-N_p_-C_α_) (7), φ(N_p_-C_α_-C_β_) (14), φ(N_m_-C_α_-C_β_) (11), φ(C_α_-C_β_-C_β_) (6), φ(C_β_-C_β_-N_t_) (6), φ(C_β_-N_t_-S) (7)	689
788 (ω_55_)	3	A_2u_	θ(C_α_-N_m_-C_α_-N_p_-C_β_) (43), θ (N_m_-C_α_-C_β_-N_t_) (45)	741
818 (ω_59_–ω_60_)	2	E_u_	r(N_t_-S) (44), φ(C_α_-N_m_-C_α_) (7), φ(N_p_-C_α_-N_m_) (8), φ(N_p_-C_α_-C_β_) (8), φ(N_t_-S-N_t_) (5)	763
843 (ω_62_–ω_63_)	11	E_u_	r(N_t_-S) (75), φ(C_β_-C_β_-N_t_) (5), φ(C_β_-N_t_-S) (10)	827
921 (ω_71_–ω_72_)	8	E_u_	r(N_p_-Ni) (5), r(C_β_-N_t_) (5), r(N_t_-S) (12), φ(C_α_-N_m_-C_α_) (12), φ(N_p_-C_α_-N_m_) (8), φ(N_m_-C_α_-C_β_) (11), φ(C_α_-C_β_-N_t_) (8), φ(C_β_-C_β_-N_t_) (6), φ(C_β_-N_t_-S) (9)	895
1161 (ω_77_–ω_78_)	100	E_u_	r(N_p_-C_α_) (41), r(N_m_-C_α_) (14), r(C_α_-C_β_) (14), r(C_β_-N_t_) (6)	1108/1109
1325(ω_84_–ω_85_)	83	E_u_	r(N_p_-C_α_) (25), r(N_p_-Ni) (5), r(C_α_-C_β_) (5), r(C_β_-C_β_) (6), r(C_β_-N_t_) (17), φ(C_α_-N_p_-C_α_) (6), φ(N_p_-C_α_-N_m_) (9), φ(N_p_-C_α_-C_β_) (6)	1269
1396 (ω_86_–ω_87_)	11	E_u_	r(N_p_-C_α_) (12), r(N_m_-C_α_) (15), r(C_α_-C_β_) (27), r(C_β_-C_β_) (20)	1347
1629 (ω_102_–ω_103_)	20	E_u_	r(N_m_-C_α_) (33), r(C_α_-C_β_) (25), r(C_β_-N_t_) (20)	1552

* Coordinates are listed provided that their contributions (shown in parentheses) are greater than ~10%. Assignment of vibrational modes based on potential energy distribution. The following designations of the coordinates are used: r—stretching of the bond; φ—bending, a change in the angle; OPB—out-of-plane bending; θ—a change in the dihedral angle.

**Table 7:** Assignment of the Raman vibrations of the H_2_TTDPz and NiTTDPz molecules.

Frequency, cm^−1^	I_rel_, %	Symmetry	Assignment *	Exp, cm^−1^
H_2_TTDPz				
223 (ω_15_)	5	A_g_	r (N_m_-C_α_) (14), r (C_α_-C_β_) (25), φ (C_α_-N_m_-C_α_) (23)	223
518 (ω_27_)	5	B_3g_	r (N_m_-C_α_) (10), r(C_β_-N_t_) (7), r (N_t_-S) (9), φ (N_p_-C_α_-C_β_) (9), φ (C_α_-C_β_-C_β_) (24), φ (C_α_-C_β_-N_t_) (13)	576/504
714 (ω_47_)	10	A_g_	r (N_p_-C_α_) (12), r (N_m_-C_α_) (12), φ (C_α_-N_m_-C_α_) (31)	697
733 (ω_49_)	10	A_g_	r (N_m_-C_α_) (11), φ (C_α_-N_p_-C_α_) (23), φ (N_p_-C_α_-C_β_) (24), φ (N_m_-C_α_-C_β_) (14)	711
855 (ω_67_)	6	A_g_	r (C_α_-C_β_) (8), r(C_β_-N_t_) (9), r(N_t_-S) (17), φ(C_β_-N_t_-S) (21), φ(N_t_-S-N_t_) (9)	823
1239 (ω_80_)	33	B_3g_	r (N_p_-C_α_) (49), r (C_α_-C_β_) (18)	1177/1158
1293 (ω_83_)	3	A_g_	r (N_p_-C_α_) (19), r(C_β_-C_β_) (18), r(C_β_-N_t_) (11), φ (C_α_-N_p_-C_α_) (10), (N_p_-C_α_-N_m_) (10)	1254/1237
1400 (ω_90_)	34	A_g_	r (C_α_-C_β_) (22), r(C_β_-C_β_) (42)	1336
1495 (ω_93_)	21	A_g_	r (C_α_-C_β_) (19), r(C_β_-N_t_) (60)	1429/1443
1595 (ω_99_)	12	A_g_	r (N_m_-C_α_) (44), r (C_α_-C_β_) (30), r(C_β_-N_t_) (10)	1532
1657 (ω_106_)	100	A_g_	r (N_m_-C_α_) (85)	1555
NiTTDPz				
253 (ω_17_)	7	A_1g_	r (N_p_-Ni) (30), r (N_m_-C_α_) (7), r (C_α_-C_β_) (26), φ (C_α_-N_m_-C_α_) (12)	
735 (ω_50_)	10	A_1g_	r (N_p_-C_α_) (7), r (N_m_-C_α_) (6), r (N_t_-S) (9), φ (C_α_-N_m_-C_α_) (20), φ (N_p_-C_α_-N_t_) (8), φ(C_β_-N_t_-S) (17), φ(N_t_-S-N_t_) (20)	709
755 (ω_51_)	11	B_1g_	r (N_p_-Ni) (13), r (N_m_-C_α_) (9), r (C_α_-C_β_) (6), (C_α_-N_p_-C_α_) (20), φ (N_p_-C_α_-C_β_) (9), φ (N_m_-C_α_-C_β_) (20)	800
844 (ω_65_)	13	A_1g_	r (N_t_-S) (76), φ(C_β_-N_t_-S) (10),), φ(C_β_-C_β_-N_t_) (5)	865
1068 (ω_74_)	4	A_2g_	r (N_p_-C_α_) (18), r (N_m_-C_α_) (34), r(C_β_-N_t_) (6), φ (C_α_ -N_m_-C_α_) (6), φ(C_β_-C_β_-N_t_) (6), φ(C_β_-N_t_-S) (7), φ(N_p_-Ni-N_p_) (5)	1024
1242 (ω_81_)	27	B_2g_	r (N_p_-C_α_) (41), r (C_α_-C_β_) (26), r (C_β_-N_t_) (10)	1182
1413 (ω_88_)	16	A_1g_	r (N_p_-C_α_) (19), r (N_m_-C_α_) (13), r (C_α_-C_β_) (10), r(C_β_-C_β_) (28)	1266
1435 (ω_89_)	48	B_1g_	r (C_α_-C_β_) (29), r(C_β_-C_β_) (40), φ(C_α_-C_β_-N_t_) (9), φ(C_β_-N_t_-S) (7)	1248
1501 (ω_92_)	15	B_1g_	r (C_α_-C_β_) (17), r(C_β_-C_β_) (9), r (C_β_-N_t_) (63)	1353
1536 (ω_94_)	7	B_2g_	r (N_p_-C_α_) (11), r (N_m_-C_α_) (41), r(C_β_-N_t_) (21), φ(N_p_-C_α_-C_β_) (8)	1362
1609 (ω_100_)	1	A_2g_	r (N_m_-C_α_) (71), r (C_α_-C_β_) (16), r(C_β_-N_t_) (5)	1534
1634 (ω_104_)	10	A_1g_	r (N_m_-C_α_) (43), r (C_α_-C_β_) (30), r(C_β_-N_t_) (6)	
1681 (ω_105_)	100	B_1g_	r (N_m_-C_α_) (84)	1572

* Coordinates are listed provided that their contributions (shown in parentheses) are greater than ~10%. Assignment of vibrational modes based on potential energy distribution. The following designations of the coordinates are used: r—stretching of the bond; φ—bending, a change in the angle; θ—a change in the dihedral angle.

## Data Availability

The data presented in this study are available on request from the corresponding author.

## Supplementary Materials

The following are available online, Figure S1: Structural formulas of porphyrazines and their derivatives, Cartesian coordinates of H_2_TTDPz optimized PBE0/pcseg-2 level of theory, Cartesian coordinates of singlet NiTTDPz optimized PBE0/pcseg-2 level of theory, Cartesian coordinates of triplet NiTTDPz optimized PBE0/pcseg-2 level of theory.

## Sample Availability

Samples of the compounds H_2_TTDPz and NiTTDPz are available from the authors.
