# Supplementary material for: Molecular Structure, Thermodynamic and Spectral Characteristics of Metal-Free and Nickel Complex of Tetrakis(1,2,5-thiadiazolo)porphyrazine
Source: Molecules. 2021 May 15;26(10):2945. doi: 10.3390/molecules26102945 (PMC8156015; doi:10.3390/molecules26102945)
Supplement: Supplementary file 1 [file molecules-26-02945-s001.zip › molecules-1204821-supplementary.pdf]

Supporting information for

«Molecular Structure, thermodynamic and spectral characteristics of metal-free and nickel complex of tetrakis(1,2,5-thiadiazole)porphyrazine»

Yuriy A. Zhabanov, Alexey V. Eroshin, Igor V. Ryzhov, Ilya A. Kuzmin, Daniil N. Finogenov and Pavel A. Stuzhin

Ivanovo State University of Chemistry and Technology, Research Institute of Chemistry of Macroheterocyclic Compounds, Sheremetievskiy av. 7, 153000 Ivanovo, Russian Federation

Content

Figure S1. Structural formulas of porphyrazines and their derivatives 2

Cartesian coordinates of H_2TTDPz optimized PBE0/pcseg-2 level of theory: 2

Cartesian coordinates of singlet NiTTDPz optimized PBE0/pcseg-2 level of theory: 3

Cartesian coordinates of triplet NiTTDPz optimized PBE0/pcseg-2 level of theory: 4

Figure S1. Structural formulas of porphyrazines and their derivatives

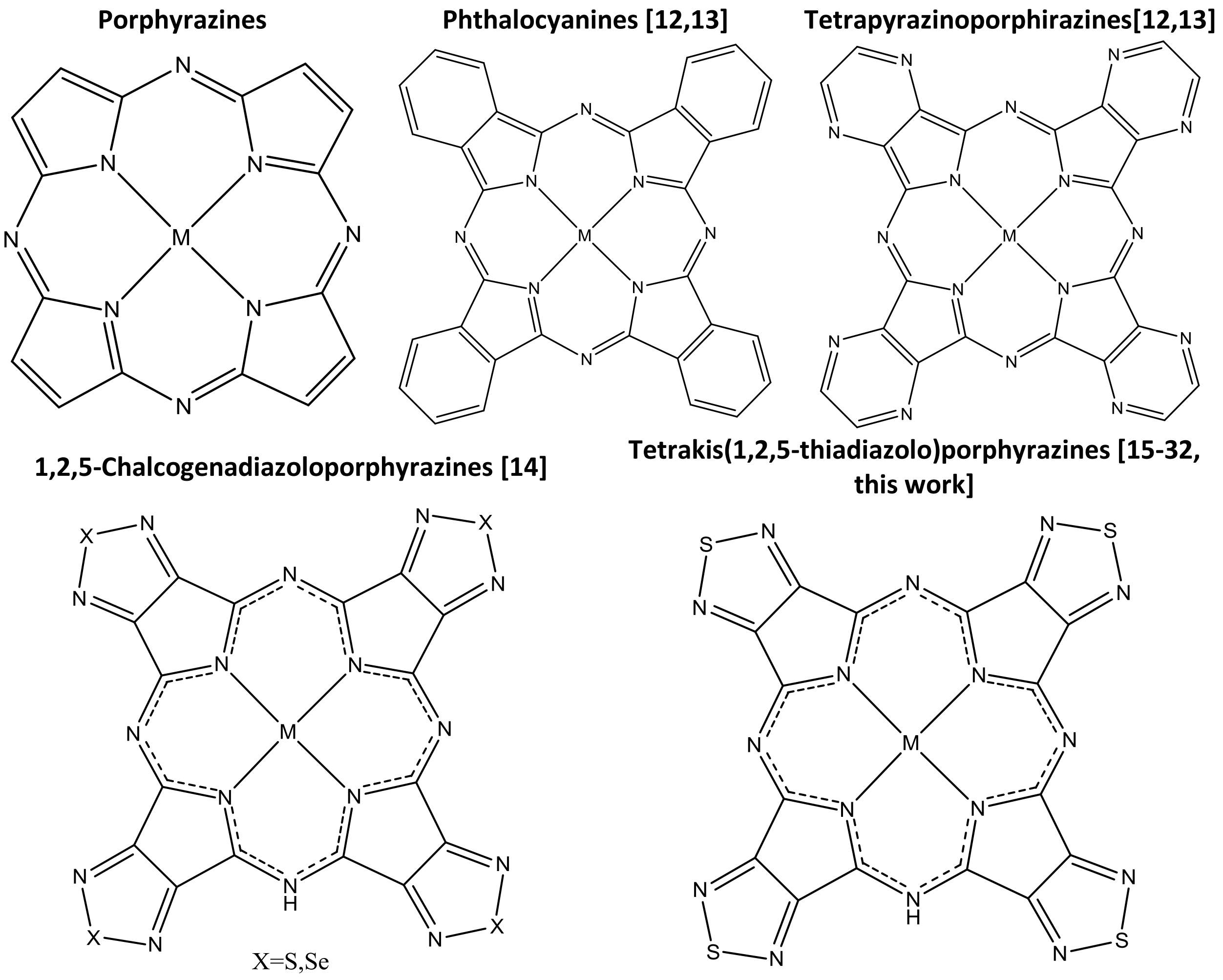

Cartesian coordinates of H_2TTDPz optimized PBE0/pcseg-2 level of theory:

N	0.000000000000	1.978811454900	0.000000000000
N	0.000000000000	-1.978811454900	0.000000000000
N	0.000000000000	0.000000000000	-2.046203960200
N	0.000000000000	0.000000000000	2.046203960200
N	0.000000000000	2.391099841400	2.374326410800
N	0.000000000000	-2.391099841400	-2.374326410800
N	0.000000000000	2.391099841400	-2.374326410800
N	0.000000000000	-2.391099841400	2.374326410800
C	0.000000000000	2.765234052400	-1.108498685100
C	0.000000000000	-2.765234052400	1.108498685100
C	0.000000000000	2.765234052400	1.108498685100
C	0.000000000000	-2.765234052400	-1.108498685100
C	0.000000000000	1.157254383600	2.788162570600
C	0.000000000000	-1.157254383600	-2.788162570600
C	0.000000000000	1.157254383600	-2.788162570600
C	0.000000000000	-1.157254383600	2.788162570600
C	0.000000000000	4.167134266100	-0.704184322900
C	0.000000000000	-4.167134266100	0.704184322900
C	0.000000000000	4.167134266100	0.704184322900
C	0.000000000000	-4.167134266100	-0.704184322900

C	0.000000000000	0.708060907000	4.163310557100
C	0.000000000000	-0.708060907000	-4.163310557100
C	0.000000000000	0.708060907000	-4.163310557100
C	0.000000000000	-0.708060907000	4.163310557100
H	0.000000000000	0.000000000000	1.035206320600
H	0.000000000000	0.000000000000	-1.035206320600
N	0.000000000000	1.253933505300	-5.362124418600
N	0.000000000000	-1.253933505300	5.362124418600
N	0.000000000000	1.253933505300	5.362124418600
N	0.000000000000	-1.253933505300	-5.362124418600
N	0.000000000000	5.358525045700	1.255864591700
N	0.000000000000	-5.358525045700	-1.255864591700
N	0.000000000000	5.358525045700	-1.255864591700
N	0.000000000000	-5.358525045700	1.255864591700
S	0.000000000000	0.000000000000	6.390694983900
S	0.000000000000	0.000000000000	-6.390694983900
S	0.000000000000	6.398911216700	0.000000000000
S	0.000000000000	-6.398911216700	0.000000000000

Cartesian coordinates of singlet NiTTDPz optimized PBE0/pcseg-2 level of theory:

N	0.000000000000	-1.926902693800	0.000000000000
N	1.926902693800	0.000000000000	0.000000000000
N	0.000000000000	1.926902693800	0.000000000000
N	-1.926902693800	0.000000000000	0.000000000000
N	-2.369204795700	-2.369204795700	0.000000000000
N	2.369204795700	-2.369204795700	0.000000000000
N	2.369204795700	2.369204795700	0.000000000000
N	-2.369204795700	2.369204795700	0.000000000000
C	1.116591026000	-2.727954576300	0.000000000000
C	2.727954576300	1.116591026000	0.000000000000
C	-1.116591026000	2.727954576300	0.000000000000
C	-2.727954576300	1.116591026000	0.000000000000
C	-1.116591026000	-2.727954576300	0.000000000000
C	2.727954576300	-1.116591026000	0.000000000000
C	1.116591026000	2.727954576300	0.000000000000
C	-2.727954576300	-1.116591026000	0.000000000000
C	0.700585082700	-4.113020594100	0.000000000000
C	4.113020594100	0.700585082700	0.000000000000
C	-0.700585082700	4.113020594100	0.000000000000
C	-4.113020594100	0.700585082700	0.000000000000
C	-0.700585082700	-4.113020594100	0.000000000000
C	4.113020594100	-0.700585082700	0.000000000000
C	0.700585082700	4.113020594100	0.000000000000
C	-4.113020594100	-0.700585082700	0.000000000000
N	1.255902817200	-5.307216104300	0.000000000000
N	5.307216104300	1.255902817200	0.000000000000
N	-1.255902817200	5.307216104300	0.000000000000
N	-5.307216104300	1.255902817200	0.000000000000
N	-1.255902817200	-5.307216104300	0.000000000000
N	5.307216104300	-1.255902817200	0.000000000000
N	1.255902817200	5.307216104300	0.000000000000
N	-5.307216104300	-1.255902817200	0.000000000000
S	0.000000000000	-6.339895711100	0.000000000000
S	6.339895711100	0.000000000000	0.000000000000
S	0.000000000000	6.339895711100	0.000000000000

S	-6.339895711100	0.000000000000	0.000000000000
Ni	0.000000000000	0.000000000000	0.000000000000

Cartesian coordinates of triplet NiTTDPz optimized PBE0/pcseg-2 level of theory:

N	-1.975864229200	0.000000000000	0.000000000000
N	0.000000000000	-1.975864229200	0.000000000000
N	1.975864229200	0.000000000000	0.000000000000
N	0.000000000000	1.975864229200	0.000000000000
N	-2.375158493000	2.375158493000	0.000000000000
N	-2.375158493000	-2.375158493000	0.000000000000
N	2.375158493000	-2.375158493000	0.000000000000
N	2.375158493000	2.375158493000	0.000000000000
C	-2.756436416700	1.118901536000	0.000000000000
C	-1.118901536000	-2.756436416700	0.000000000000
C	2.756436416700	-1.118901536000	0.000000000000
C	1.118901536000	-2.756436416700	0.000000000000
C	2.756436416700	1.118901536000	0.000000000000
C	-1.118901536000	2.756436416700	0.000000000000
C	-2.756436416700	-1.118901536000	0.000000000000
C	1.118901536000	2.756436416700	0.000000000000
C	-4.159830369900	0.703633342600	0.000000000000
C	-0.703633342600	-4.159830369900	0.000000000000
C	4.159830369900	-0.703633342600	0.000000000000
C	0.703633342600	-4.159830369900	0.000000000000
C	4.159830369900	0.703633342600	0.000000000000
C	-0.703633342600	4.159830369900	0.000000000000
C	-4.159830369900	-0.703633342600	0.000000000000
C	0.703633342600	4.159830369900	0.000000000000
N	-5.347384719300	1.255773280000	0.000000000000
N	-1.255773280000	-5.347384719300	0.000000000000
N	5.347384719300	-1.255773280000	0.000000000000
N	1.255773280000	-5.347384719300	0.000000000000
N	5.347384719300	1.255773280000	0.000000000000
N	-1.255773280000	5.347384719300	0.000000000000
N	-5.347384719300	-1.255773280000	0.000000000000
N	1.255773280000	5.347384719300	0.000000000000
S	-6.393555437300	0.000000000000	0.000000000000
S	0.000000000000	-6.393555437300	0.000000000000
S	6.393555437300	0.000000000000	0.000000000000
S	0.000000000000	6.393555437300	0.000000000000
Ni	0.000000000000	0.000000000000	0.000000000000
